# Cytokine Response to SARS-CoV-2 Infection in Children

**DOI:** 10.3390/v13091868

**Published:** 2021-09-18

**Authors:** Antonietta Curatola, Antonio Chiaretti, Serena Ferretti, Giulia Bersani, Donatella Lucchetti, Lavinia Capossela, Alessandro Sgambato, Antonio Gatto

**Affiliations:** 1 Department of Pediatrics, Università Cattolica del Sacro Cuore, 00168 Rome, Italy; c.anto91@hotmail.it (A.C.); antonio.chiaretti@policlinicogemelli.it (A.C.); laviniacapossela@gmail.com (L.C.); 2 Department of Pediatrics, Fondazione Policlinico Universitario Agostino Gemelli IRCCS, 00168 Rome, Italy; giuliabersani1@gmail.com (G.B.); antonio.gatto@policlinicogemelli.it (A.G.); 3 Department of Translational Medicine and Surgery, Faculty of Medicine, Catholic University of the Sacred Heart, 00168 Rome, Italy; donatella.lucchetti@unicatt.it; 4 Centro di Riferimento Oncologico della Basilicata (IRCCS-CROB), 05028 Rionero in Vulture (PZ), Italy; alessandro.sgambato@unicatt.it

**Keywords:** children, cytokines, COVID-19, IL-6, TNF-α

## Abstract

The causal connection between serum biomarkers and COVID-19 severity or pathogenicity in children is unclear. The aim of this study was to describe clinical and immunological features of children affected by COVID-19. The secondary aim was to evaluate whether these cytokines could predict severity of COVID-19. All children (aged 0−18) admitted to the Pediatric Emergency Department and tested with nasopharyngeal swab for SARS-CoV-2 were recruited and assigned to three groups: COVID-19, other infections, control group. Clinical and laboratory data of these patients, including circulating cytokine levels, were analyzed in three groups. Fever was the most frequent symptom in COVID-19 (67.3%). Neutropenia was found in the COVID-19 group (*p* < 0.05); no difference was observed for lymphocyte counts in the three groups. Higher levels of IL-6 and TNF-alpha were found in the COVID-19 group compared to other infections and control groups (*p* = 0.014 and *p* = 0.001, respectively). Whereas, in the COVID-19 group, no difference was observed as for the same cytokines among sub-groups of different disease severity (*p* = 0.7 and *p* = 0.8). Serum levels of IL-6 and TNF-alpha were higher in COVID-19 children than in children with other infectious diseases, but those levels did not correlate with disease severity. Clinical studies in a large pediatric population are necessary to better define the role of the immune-mediated response in SARS-CoV-2 infections in children.

## 1. Introduction

Since the end of 2019, the Severe Acute Respiratory Syndrome Coronavirus-2 (SARS-CoV-2) has spread around the world causing the devastating Coronavirus Disease 2019 (COVID-19) pandemic. Despite very high numbers of infected persons and deaths, after several months, COVID-19 still hides aspects regarding the pathogenesis and inter-individual variability of clinical manifestations. Furthermore, childhood data of the disease are even more limited, especially as for its clinical course, which differs from that in adults. Despite different underlying and comorbid conditions affecting children, unlike other viruses, SARS-CoV-2 seems to be less contagious and causes a less severe course in children worldwide [1,2,3,4,5,6]. In addition, severe complications are also less frequent and severe in children compared to adults affected with COVID-19, and there is no explanation for this difference [7,8,9,10,11].

The causal connection between biomarkers and COVID-19 severity or pathogenicity is currently unclear. However, the ‘cytokine storm’, which is an excessive immune response to the infection, is associated with widespread tissue damage and high cytokine production [12]. Different studies have detected cytokine expression in patients with moderate/severe disease. Thus, suppressing the cytokine storm is fundamental to improving the clinical condition of patients [13]. However, the knowledge of these data is wider for adult patients, but only few studies investigate biomarkers expressed in children with COVID-19 [14,15].

The main aim of this study was to describe clinical and immunological features of children affected by COVID-19. The secondary aim was to evaluate whether these cytokines could predict severity of COVID-19 disease.

## 2. Materials and Methods

### 2.1. Patients and Methods

We conducted a prospective, observational, clinical study including all children, aged between 0 and 18 years, admitted to the Pediatric Emergency Department (PED) with COVID-19 symptoms plus nine children admitted to the PED for non-infectious diseases, voluntarily enrolled as controls. Based on clinical features of patients and microbiological results of nasopharyngeal swabs, the study population was divided into 3 groups: Group 1, children affected by COVID-19; Group 2, children with other viral infectious diseases; Group 3, children without infectious diseases (controls).

All children were evaluated with a nasopharyngeal swab for SARS-CoV-2 infection according to our hospital internal protocol. The study was conducted between May 2020 and March 2021 at the Fondazione Policlinico Universitario A. Gemelli IRCCS, Rome, which is a tertiary referral hospital. Written informed consent was obtained from caregivers of all participants. The study was approved by the Ethics Committee (prot. 0018501/20).

Data regarding demographic and clinical characteristics of enrolled children, as well as values of routinely performed laboratory tests and imaging, were collected from medical records. Since the beginning of the COVID-19 outbreak, all possible COVID-19 cases were tested for a complete blood count and C-reactive protein (CRP) levels, glutamic-pyruvic transaminase (GPT), lactate dehydrogenase (LDH), creatine phosphokinase (CPK), fibrinogen, and D-dimer. Serum samples were collected and stored at −80 °C for cytokine quantification. Regarding imaging, chest X-rays were performed only in the presence of respiratory symptoms.

According to the criteria of Parri et al., COVID-19 infection in children was classified as mild, moderate, severe, or critical. These criteria included the presence of respiratory signs and symptoms (upper respiratory tract infection, cough, difficult breathing, apnea) or any other danger signs (convulsions, lethargy, severe dehydration, hypotonia), a radiological diagnosis of pneumonia from a chest X-ray or computerized tomography, or admission to the Pediatric Intensive Care Unit [16].

The diagnosis of SARS-CoV-2 was confirmed at admission to PED using a real-time reverse transcriptase polymerase chain reaction (RT-PCR) method on nasopharyngeal swabs. A Luminex xMAP system (Bio-Plex 200 System, Bio-Plex Manager Software 6.0, Bio-Rad Laboratories, Hercules, CA, USA), which is a multiplex ELISA-based immunoassay, was used to assess the levels of circulating cytokines. We used the Human Magnetic Luminex Screening Assay (BioTechne, Milano, Italy) which allows the simultaneous detection of the following circulating analytes: CD163, CXCL9, IL1β, S100b, cd25, IFNγ, TNF-α, and IL6.The assay was performed according to manufacturers’ instructions, and the concentrations were calculated by a software provided by the manufacturer using the standard curve (Bio-Plex Manager Software, Bio-Rad Laboratories, Hercules, CA). Serum samples were assayed in duplicate, and hemolyzed samples were removed from analysis according to vendor recommendations. We decided to test these cytokines as recognized reliable biomarkers in disorders that have inflammatory processes as a common basis and for their involvement in the “cytokine storm” [13,14,15].

### 2.2. Statistical Analysis

Categorical variables were reported as counts and percentages. Continuous variables were expressed as means and standard deviations or as median and inter-quartile ranges (IQR), if not normally distributed. Statistical comparisons between groups were obtained for categorical variables by Chi-squared tests or Fisher’s exact test, as appropriate. Kruskal–Wallis tests were used for intragroup comparisons for continuous variables not normally distributed, and ANOVA tests were used for normally distributed variables. The Bonferroni correction was applied for post hoc analysis. The other statistical analyses were performed using Mann–Whitney tests and Student’s *t*-tests, as appropriate. A multivariate logistic regression model was used to evaluate which determinants were independently associated to the diagnosis of COVID-19 disease in children. Logistic regression results are reported as odds ratio (OR) with 95% confidence intervals. Two-sided *p* values <0.05 were considered statistically significant. All data analyses were performed using the Statistical Package for the Social Sciences (SPSS for Windows, version 25.0, SPSS Inc., Chicago, IL, USA).

## 3. Results

### 3.1. Patients: Clinical and Laboratory Features

During the study period, 70 children admitted to our PED were tested for SARS-CoV2, and blood sampling for cytokines assay was performed. Eighteen of them were excluded due to hemolyzed blood samples, and 52 were included in the study. None of the patients presented heart disease, whereas immune deficiency was observed in three (5.8%) children. The median age of enrolled children was 5.46 years (IQR 2.2–12.4), and 28 (53.8%) were males. Among enrolled patients, 18 (34.6%) children had a COVID-19-positive contact, while 34 (65.4%) had no contact. Out of the total sample, 8 (15.4%) were asymptomatic at diagnosis, while 44 (84.6%) presented various symptoms including fever and/or respiratory, gastrointestinal, and/or neurological signs or symptoms. Specifically, 35 (67.3%) had fever, 14 (26.9%) had respiratory signs/symptoms, and 14 (26.9%) presented gastroenterological symptoms (abdominal pain, vomit, and diarrhea); neurological symptoms, such as convulsion, irritability, headache, anosmia/ageusia, were observed in 23 (44.2%) patients. Non-specific flu-like symptoms, such as muscular–articular pains and asthenia, were reported in 12 (23.1%) and 18 (34.6%) patients, respectively. In addition, 7 (13.5%) children presented a skin involvement characterized by polymorphous rash.

According to clinical features and results of nasopharyngeal swabs, 27 (51.9%) patients were affected by COVID-19 (Group 1), 16 (30.8%) COVID-19 negative but with other infectious diseases (Group 2), and 9 (17.3%) without infectious diseases (Group 3).

No difference was observed for gender and cardiac disease or immune deficiency in different groups. A history of COVID-19-positive contact was strongly associated with an increased risk of positive swab (χ2 (2, *N* = 52) = 20.31, *p* < 0.01; Cramer’s V = 0.62). No differences were observed in the three groups for respiratory, gastroenterological, neurological, and non-specific flu-like symptoms. Fever was more frequent in children with COVID-19 than in those with other infectious diseases (χ2 (2, *N* = 52) = 27.25, *p* < 0.01; Cramer’s V = 0.72) (Table 1).

In the COVID-19 group, the patients were classified as mild, moderate, severe, and critical according to Parri et al.’s clinical criteria. Four (7.7%) patients were asymptomatic at diagnosis, 18 (34.6%) were diagnosed as mild COVID-19 patients, and 4 (7.7%) were moderate COVID-19 patients. Only one (1.9%) child developed severe COVID-19; the patient was a 4-month-old male who developed Multisystem Inflammatory Syndrome (MIS-C) 3 days after hospitalization and was treated with intravenous corticosteroids and immunoglobulins according to American guidelines [17].

Regarding the laboratory features, we observed that neutropenia was significantly more frequent in the SARS-CoV-2-positive group. The median neutrophil (N) count of COVID-19-positive children was statistically different compared with children with other infectious and non-infectious diseases (*p* < 0.05). No statistically significant difference was found for white blood cell (WBC), platelet (PLT) and lymphocyte (L) counts, hemoglobin (Hb), PCR, GPT, CPK, LDH, fibrinogen, and D-dimer.

In addition, only 18 (34.6%) children of the total sample underwent a chest X-ray, of which 4 (22.3%) were in Group 1 and 3 (16.6%) in Group 2 (*p* = 0.54). No patient underwent a chest CT scan. The rate of hospitalization was also not significantly different among the three groups (59.3% Group 1 vs. 62.5% Group 2 vs. 22.2% Group 3, *p* = 0.13).

### 3.2. Cytokine Screening

**Figure 1:**
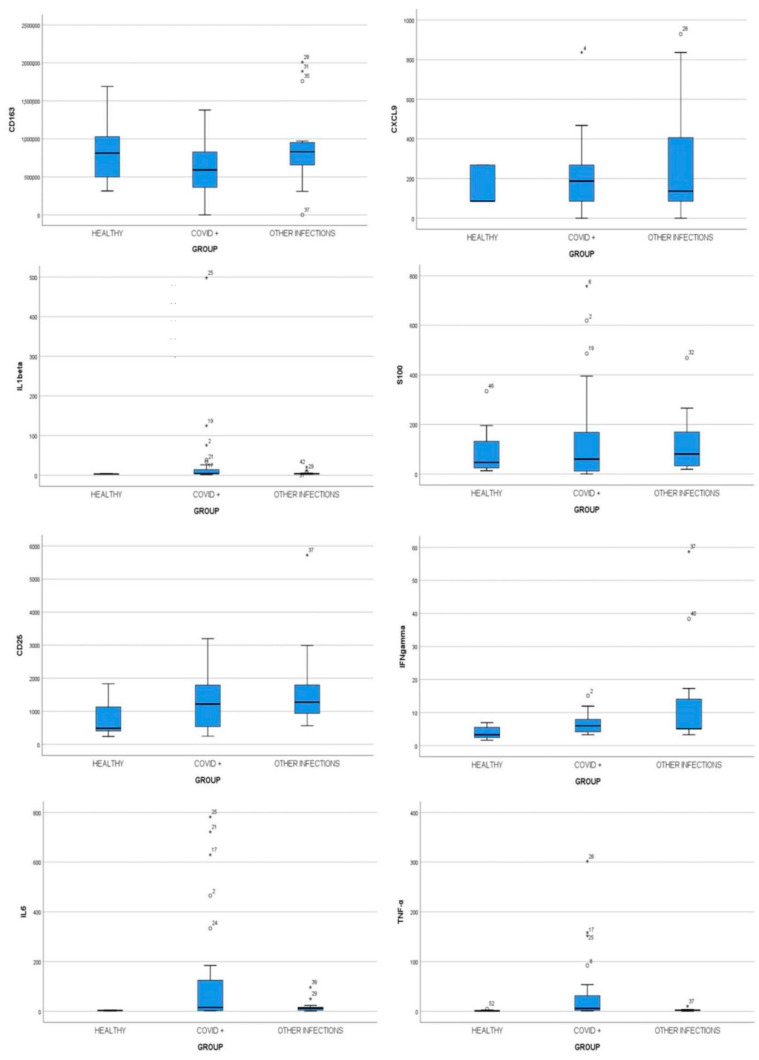
Cytokines evaluation in children affected by COVID-19 (Group 1), with other infectious diseases (Group 2), and children without infections (Group 3). Plasmatic levels of CD163, CXCL9, IL1β, S100b, CD25, IFN-γ, TNF-α, and IL6 were measured by Bead Array flow cytometry assay on a single serum sample at admission to the ED using the Human Magnetic Luminex Screening Assay. ANOVA and Kruskal–Wallis comparison tests were used to compare values between COVID-19 children, children with other infections, and healthy control subjects.

**Figure 2:**
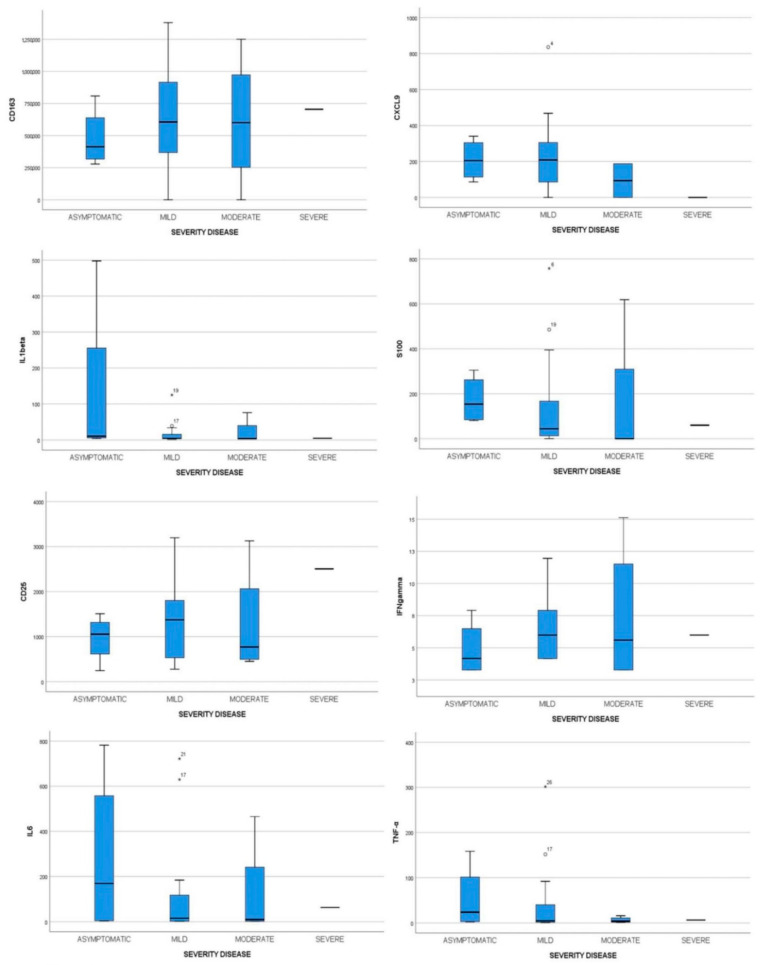
Cytokines evaluation in children affected by COVID-19 with different severity disease: asymptomatic, mild, moderate, and severe. Plasmatic levels of CD163, CXCL9, IL1β, S100b, CD25, IFN-γ, TNF-α, and IL6 were measured by Bead Array flow cytometry assay on a single serum sample. ANOVA and Kruskal–Wallis comparison tests were used to compare values between different COVID-19 severities.

Immunological features of 52 children were studied to detect possible associations with COVID-19 disease and its severity. Cytokine profiling was performed with sera of patients at time of diagnosis, corresponding to the early stage of disease. Specifically, we analyzed in all patients the following cytokines: CD163, CXCL9, IL-1β, S100b, cd25, IFNγ, TNFalfa, and IL6 and compared their levels in the three groups. No statistically significant difference was observed for CD163, CXCL9, IL-1β, and S100b. Higher levels of IL-6 and TNF-α were found in samples of Group 1 compared to other infections and control groups (*p* = 0.014 and *p* = 0.001, respectively) (Figure 1). However, in the COVID-19 group no difference was observed as for the same cytokines among sub-groups of different disease severity (*p* = 0.736 and *p* = 0.781, respectively). The complete cytokine profile of COVID-19 severity subgroups is shown in Table 2 and Figure 2.

In addition, among three groups statistically significant differences were found for IFN-γ and CD25 (*p* < 0.05), but the median value of these cytokines was higher in subjects with other infectious diseases, Group 2 (Table 3).

Analyzing cytokine levels in the different groups, we observed that in Group 1 some patients had extremely high values of IL-1β, CD25, IFN-γ, IL6, and TNF-α. We therefore created a subgroup comprising these patients that we classified as “COVID-plus”. Comparing the levels of these cytokines in the COVID-plus group with the other three groups, statistically significant differences were found for IL-1β, CD25, IFN-γ, IL-6, and TNF-α (*p* < 0.05).

According to Parri et al.’s classification of disease severity, in the COVID-plus group we identified two (18.2%) asymptomatic children, seven (63.6%) with mild symptoms, and two (18.2%) with moderate symptoms. No statistically significant differences were observed as for severity disease into two COVID-19 groups (COVID vs. COVID-plus).

Multivariate logistic regression analysis was performed to ascertain the effects of age, IL-6, and TNF-α levels on the likelihood that participants had COVID-19. The logistic regression model was statistically significant, χ2 (3) = 17.24, *p* < 0.05. The analysis showed no independent predictor of COVID-19.

## 4. Discussion

The main aim of this study was to describe clinical and immunological features of children affected by COVID-19. According to literature, fever was the symptom most frequently associated with SARS-CoV-2 infection in children, even if in this population COVID-19 clinical presentation can be extremely variable, including respiratory, gastrointestinal, neurological, or cutaneous signs and symptoms, alone or in combination with one another [18].

As regard to laboratory features, in our study population neutropenia was significantly more frequent in the SARS-CoV-2-positive group. Instead, no statistically significant difference was observed for lymphocyte counts in the three groups. In addition, no statistically significant associations were observed between COVID-19 disease and other available laboratory parameters, such as inflammation parameters (PCR), LDH, CPK, GPT, fibrinogen, and D-dimer.

From serum analysis, we found that the levels of IL-6 and TNF-α on admission time were higher in children with COVID-19 compared to the other groups, in line with previous studies performed on adults [19,20]. IL-6 is an important proinflammatory cytokine with different inflammatory roles; it was produced by various cells, i.e., macrophages, neutrophils, dendritic cells, and lymphocytes, and promotes the differentiation of naïve CD4 T cells into effector and helper cells [19]. TNF-α has different functions, such as to promote the expression of genes for growth factors, other cytokines, transcription factors, and receptors [19]. All these factors are involved in the inflammation process in response to a pathogen.

In addition, from the analysis of cytokines in our patients we identified a subgroup, called COVID-plus, including COVID-19-positive patients who presented much higher levels of IL-6, TNF-α, IL-1β, and CD25 than other groups.

The secondary aim was to evaluate whether these cytokines could predict severity of COVID-19 disease. For this reason, we divided children in four groups of differing disease severity (mild, moderate, severe, and critical), according to Parri et al.’s classification. However, no correlation was found between analyzed cytokines and the clinical disease severity in our patients, according to Qian et al. [21]. This could be due to the small sample studied but also because these cytokines were only assayed at an early stage of the disease, on admission to the ED.

According to our results, in other pediatric studies, and also in studies performed on adults, the main risk factor associated with COVID-19 disease was a history of contact with a COVID-19 case, and fever was the most frequent symptom [22,23]. Lazzerini et al., in a study performed with a pediatric population, stated that the main condition detected in the laboratory analyses was neutropenia [18]. However, lymphopenia does not seem to be associated with COVID-19 disease in children, in contrast to studies in adults in which lymphocyte counts would also be related to the severity of COVID-19 disease and the number of viral copies from nasopharyngeal swabs [21,22,23,24]. Therefore, unlike in adults, in children there are few clinical and laboratory features to help us distinguish patients with SARS-CoV-2 infection from those with other infectious conditions. This may be due to the milder initial clinical presentation of this disease in pediatric age. Additionally, the lack of a correlation between a particular cytokine and the COVID-19 disease severity and the predominant mild clinical course of COVID-19 disease in children may be due to the reduced inflammatory response to SARS-CoV-2 infection that instead was described in adults [20].

Cytokines are important mediators of the inflammatory response and play a central role in the pathophysiology of COVID-19. Different studies were performed in adult populations affected by COVID-19 to evaluate the disease severity, but very little is known about the pediatric population. Jamillaux et al. stated that some cytokines, such as type-I interferon and interleukin-7, were protective for patients with COVID-19 disease, while others, L-1β, IL-6, and TNF-α, could be harmful, especially during the so-called “cytokine storm” [25]. Ruan et al., in a study on adult samples, showed that critical patients admitted to the Intensive Care Unit presented higher levels of IL-2, IL-7, IL-10, granulocyte-colony-stimulating factor, IP-10, monocyte chemoattractant protein-1 (MCP-1), macrophage inflammatory protein-1A (MIP-1A), and tumor necrosis factor-α (TNF-α) [26]. Moreover, different studies have identified IL-6 as the indicator of disease severity in adults with COVID-19 infections [19,26]; in particular, non-survival in adults with respiratory distress syndrome (ARDS) was linked to IL-6 and IL-1 increases [20,27]. In regard to cytokine response in children with COVID-19, few data are available in the literature. Some authors, unlike our reports, stated that pediatric COVID-19 cases with mild clinical presentation had IL-6 levels within a normal range or rarely elevated [15,28]. Moratto et al. described in a small case series that IL-6 concentrations were higher in sera of adults with COVID-19, whereas IL-1β was significantly higher in COVID-19 children [29].

Moreover, Aceti et al. demonstrated that S100B concentration was associated with inflammation markers (ferritin, C-reactive protein, procalcitonin) and organ damage markers (alanine aminotransferase, creatinine) in adults, but in our study we did not find it in pediatric patients, probably because we included prevalently mild cases of COVID-19 disease [30].

Although our study contributes to improving knowledge of the cytokine cascade triggered by COVID-19 in the pediatric population, it presented some limitations. First, it is a prospective single-center study of children admitted to PED; therefore, the small number of COVID-19-positive patients enrolled may mask small differences in cytokine levels between groups. Second, we included very few cases of severe/critical disease in which a major cytokine response could be observed. Third, we only collected sera from these patients on admission and did not provide for subsequent checks over time because most of them were mild cases discharged at home. Finally, we did not study the late consequences of COVID-19 infection in this pediatric population to assess whether there was a correlation between the levels of some cytokines and late outcomes. Nonetheless, the strength of this study is that, to our knowledge, it is the first pediatric study that describes these cytokines responses, CD163, CXCL9, IL1β, S100b, cd25, IFNγ, TNFα, and IL6, to COVID-19 infection in children. Subsequent studies, performed in large numbers, will be needed to assess the correlation between these cytokines and disease severity before these cytokines can be used as diagnostic markers.

## 5. Conclusions

In our cohort, we observed that serum levels of IL-6 and TNF-α were more elevated at diagnosis in children affected by COVID-19 compared with other infectious diseases. However, these cytokines levels did not correlate with disease severity, probably because in our small cohort mainly mild cases were enrolled and analyzed. These findings support the need for future studies with larger cohorts to better define the role of the immune-mediated response in SARS-CoV-2 infection.

## Figures and Tables

**Table 1:** Characteristics and clinical symptoms of the study population (*N* = 52).

	Group 1 (*n* = 27)	Group 2 (*n* = 16)	Group 3 (*n* = 9)	*p*-Value
Gender (M)	16 (17.1%)	7 (25.0%)	5 (17.9%)	0.567
Contact with COVID-19 positive	17 (63.0%)	1 (6.3%)	0 (0.0%)	0.000
Cardiopathy	0 (0.0%)	0 (0.0%)	0 (0.0%)	-
Immune deficiency	1 (3.7%)	2 (12.5%)	0 (0.0%)	0.570
Tachycardia	2 (7.4%)	2 (12.5%)	0 (0.0%)	0.640
Tachypnea	0 (0.0%)	0 (0.0%)	0 (0.0%)	-
Fever	19 (70.4%)	16 (100.0%)	0 (0.0%)	0.000
Cough	7 (25.9%)	5 (31.3%)	1 (11.1%)	0.570
Rinitis	4 (14.8%)	0 (0.0%)	0 (0.0%)	0.280
Dyspnea	1 (3.7%)	0 (0.0%)	0 (0.0%)	1.000
Respiratory symptoms	8 (29.6%)	5 (31.3%)	1 (11.1%)	0.588
Rash	2 (28.6%)	4 (57.1%)	1 (14.3%)	0.292
Muscle aches	6 (22.2%)	4 (25.0%)	2 (22.2%)	1.000
Chest pain	3 (11.1%)	0 (0.0%)	1 (11.1%)	0.377
Abdominal Pain	4 (14.8%)	6 (37.5%)	1 (11.1%)	0.232
Vomit	2 (7.4%)	2 (12.5%)	1 (11.1%)	0.838
Diarrhea	3 (11.1%)	2 (12.5%)	0 (0.0%)	0.692
Gastrointestinal symptoms	6 (22.2%)	7 (43.8%)	1 (11.1%)	0.172
Headache	2 (7.4%)	2 (12.5%)	1 (11.1%)	0.838
Asthenia	8 (29.6%)	7 (43.8%)	3 (33.3%)	0.684
Seizures	0 (0.0%)	0 (0.0%)	0 (0.0%)	-
Ageusia	0 (0.0%)	0 (0.0%)	0 (0.0%)	-
Anosmia	2 (7.4%)	0 (0.0%)	0 (0.0%)	0.674
Neurological symptoms	11 (40.7%)	8 (50.0%)	4 (44.4%)	0.929
Lymphadenopathy	3 (11.1%)	3 (18.8%)	0 (0.0%)	0.448
Pharyngodynia	4 (14.8%)	5 (31.3%)	0 (0.0%)	0.140
Conjunctivitis	0 (0.0%)	0 (0.0%)	0 (0.0%)	-
Asymptomatic	4 (14.8%)	0 (0.0%)	4 (44.4%)	0.120
Hospitalization	16 (59.3%)	10 (62.5%)	2 (22.2%)	0.131

Data expressed as total numbers and percentage (%); Group 1: children affected by COVID-19; Group 2: febrile children affected by other viral infections; Group 3: children without infections.

**Table 2:** Cytokine profiles in COVID-19 children with different severity disease.

Cytokine	Asymptomatic (*n* = 4)	Mild (*n* = 18)	Moderate (*n* = 4)	Severe (*n* = 1)	*p*-Value
CD163 (pg/mL)	477937.53 ± 233631.34	630800.59 ± 367831.35	612994.86 ± 516348.23	704946.15	0.893
CXCL9 (pg/mL)	204.70 (86.16–340.34)	208.48 (0.00–836.24)	93.72 (0.00–187.44)	0.00	0.246
IL-1β (pg/mL)	10.17 (3.69–497.80)	4.21 (1.86–124.93)	3.34 (2.34–75.62)	4.39	0.511
S100 (pg/mL)	153.75 (80.73–304.62 )	44.05 (0.00–757.85)	0.00 (0.00–618.56)	60.03	0.254
CD25 (pg/mL)	1052.83 (245.49–1507.56)	1372.44 (276.29–3195.35)	768.19 (452.14–3128.03)	2505.33	0.458
IFN-γ (pg/mL)	4.17 (3.28–7.91)	5.99 (4.16–11.95)	5.59 (3.28–15.12)	5.99	0.639
IL-6 (pg/mL)	169.65 (3.35–781.95)	15.30 (1.39–721.59)	9.82 (1.70–465.22)	63.25	0.736
TNF-α (pg/mL)	23.75 (2.51–158.30)	4.72 (0.57–301.59)	3.90 (1.44–15.96)	6.42	0.781

**Table 3:** Cytokine profiles in COVID-19 children (Group 1), children with other infectious (Group 2), and children without infectious diseases (Group 3).

Cytokine	Group 1 (*n* = 27)	Group 2 (*n* = 16)	Group 3 (*n* = 9)	*p*-Value
CD163 (pg/mL)	608262.46 ± 358962.97	922327.12 ± 541792.5	805893.37 ± 434707.79	0.074
CXCL9 (pg/mL)	187.44 (86.16–277.76)	136.8 (86.16–406.25)	86.16 (86.16–268.57)	0.705
IL-1β (pg/mL)	4.39 (3.17–15.5)	3.86 (3.25–5.19)	3.00 (2.50–4.21)	0.057
S100 (pg/mL)	60.03 (10.05–169.41)	80.70 (31.77–172.99)	46.77 (13.1–195.59)	0.663
CD25 (pg/mL)	1219.55 (532.17–1802.42)	1274.53 (928.01–1926.75)	484.53 (370.07–1132.12)	0.039
IFN-γ (pg/mL)	5.06 (4.16–7.91)	5.99 (5.06–14.58)	3.28 (2.05–5.76)	0.044
IL-6 (pg/mL)	15.3 (2.67–131.7)	12.29 (4.46–17.27)	3.01 (1.50–4.41)	0.014
TNF-α (pg/mL)	5.67 (1.84–40.18)	2.38 (1.31–2.93)	0.81 (0.81–2.11)	0.001

## Data Availability

Not applicable.
